# Inhibition of Mdmx (Mdm4) *in vivo* induces anti-obesity effects

**DOI:** 10.18632/oncotarget.23837

**Published:** 2018-01-02

**Authors:** Ning Kon, Donglai Wang, Tongyuan Li, Le Jiang, Li Qiang, Wei Gu

**Affiliations:** ^1^ Institute for Cancer Genetics, College of Physicians and Surgeons of Columbia University, New York, New York, USA; ^2^ Naomi Berrie Diabetes Center, Department of Medicine, College of Physicians and Surgeons of Columbia University, New York, New York, USA; ^3^ Department of Pathology and Cell Biology, College of Physicians and Surgeons of Columbia University, New York, New York, USA

**Keywords:** p53, Mdmx, Mdm4, metabolism, adipose tissues

## Abstract

Although cell-cycle arrest, senescence and apoptosis remain as major canonical activities of p53 in tumor suppression, the emerging role of p53 in metabolism has been a topic of great interest. Nevertheless, it is not completely understood how p53-mediated metabolic activities are regulated *in vivo* and whether this part of the activities has an independent role beyond tumor suppression. Mdmx (also called Mdm4), like Mdm2, acts as a major suppressor of p53 but the embryonic lethality of mdmx-null mice creates difficulties to evaluate its physiological significance in metabolism. Here, we report that the embryonic lethality caused by the deficiency of *mdmx*, in contrast to the case for *mdm2*, is fully rescued in the background of *p53*^*3KR/3KR*^, an acetylation-defective mutant unable to induce cell-cycle arrest, senescence and apoptosis. *p53*^*3KR/3KR*^*/mdmx*^*-/-*^ mice are healthy but skinny without obvious developmental defects. *p53*^*3KR/3KR*^*/mdmx*^*-/-*^ mice are resistant to fat accumulation in adipose tissues upon high fat diet. Notably, the levels of p53 protein are only slightly increased and can be further induced upon DNA damage in *p53*^*3KR/3KR*^*/mdmx*^*-/-*^ mice, suggesting that Mdmx is only partially required for p53 degradation *in vivo*. Further analyses indicate that the anti-obesity phenotypes in *p53*^*3KR/3KR*^*/mdmx*^*-/-*^ mice are caused by activation of lipid oxidation and thermogenic programs in adipose tissues. These results demonstrate the specific effects of the p53/Mdmx axis in lipid metabolism and adipose tissue remodeling and reveal a surprising role of Mdmx inhibition in anti-obesity effects beyond, commonly expected, tumor suppression. Thus, our study has significant implications regarding Mdmx inhibitors in the treatment of obesity related diseases.

## INTRODUCTION

Tumor suppressor p53 is a transcription factor, exerting its regulations on gene transcription through direct binding to p53 responsive elements and recruiting cofactors to facilitate assembly of transcription machinery [1, 2]. Extensive studies have revealed that p53 transcriptional activities are highly versatile, affecting a large number of genes and in a wide range of functions both in basal and stress conditions, including fertility, DNA damage, as well as oxidative and metabolic stress [3, 4]. The versatility of p53 is in part owing to its posttranslational modifications (PTMs) [5-7]. As one of the important modifications, acetylation has been shown to be critical for regulation of p53 transcriptional activities [8]. Specifically, our previous studies reveal that acetylation of lysines in the p53 DNA binding domain is indispensable for the transcriptional program to induce apoptosis, senescence, and cell cycle arrest. Combined mutations of p53 K117, K161, and K162 to arginine (3KR) in mouse result in loss of induction of apoptosis, senescence, and cell cycle arrest by the mutant p53 3KR protein [9]. Moreover, *p53*^*3KR/3KR*^ mice do not show early-onset tumor formation, suggesting that additional p53 dependent functions play important roles in tumor suppression [9].

Ubiquitination is another important posttranslational modification of p53, which is mainly controlled by p53 E3 ligase Mdm2, and Mdm2-related protein Mdmx [10-16]. Both *mdm2* and *mdmx* knockout mice are embryonic lethal, due to activation of p53 and subsequent induction of apoptosis, senescence and growth arrest. Importantly, the lethality of *mdm2* and *mdmx* knockout mice are rescued by concomitant deletion of *p53*, suggesting that Mdm2 and Mdmx exert their functions mainly through negative regulation of p53 [17-19]. However, the embryonic lethality of *mdm2* and *mdmx* knockout mice limits our understanding of p53 functions beyond cell death and control of cell cycle. Interestingly, the embryonic lethality of *mdm2* and *mdmx* knockout mice can be rescued by mutant p53 with reduced functions and by genetically modified p53 with restricted p53 expression, allowing studies of Mdm2 and Mdmx-dependent temporal and tissue specific phenotypes in those mice [20-22]. Furthermore, p53 is activated through dissociation with Mdmx mediated by phosphorylation of Mdmx under metabolic stress, highlighting the importance of mdmx in p53-dependent metabolic regulation [23].

In light of these studies, and particularly the absence of induction of apoptosis, senescence and cell cycle arrest in mutant *p53*^*3KR/3KR*^ mice, we reason that the combination of p53 3KR mutation and the absence of p53 negative regulator Mdmx may provide unique opportunities to uncover novel p53-regulated functions, particularly in metabolic regulations, which would be otherwise masked by cell death and cell growth arrest in wild-type *p53* mice.

Notably, our previous study showed that the embryonic lethality caused by the deficiency of *mdm2* was only partially rescued in the background of *p53*^*3KR/3KR*^. We also observed high levels of p53 protein and activation of p53-mediated ferroptosis associated with loss of Mdm2 in *p53*^*3KR/3KR*^*/mdm2*^*-/-*^ mice. In this study, we report that the *p53 3KR* mutation fully rescued the embryonic lethality of *mdmx* knockout mice and that p53 protein levels were only slightly increased in *p53*^*3KR/3KR*^*/mdmx*^*-/-*^ mice. Interestingly, *p53*^*3KR/3KR*^*/mdmx*^*-/-*^ mice displayed significant reduction of body fat and increase of energy expenditure, compared to *p53*^*3KR/3KR*^ littermates. In addition, *p53*^*3KR/3KR*^*/Mdmx*^*-/-*^ mice were protected from obesity and insulin resistance upon high fat diet treatment. We further demonstrated that the metabolic benefits were achieved through brown remodeling of white adipose tissues. To explore the mechanism leading to the anti-obesity phenotype, we identified *ELOVL3* as a bona fide p53 target gene and a key mediator of the browning effects in *p53*^*3KR/3KR*^*/mdmx*^*-/-*^ mice. Taken together, by abolishing p53-dependent apoptosis, senescence, and cell cycle arrest through *p53 3KR* mutations, we uncovered a catabolic function of p53 in adipose biology.

## RESULTS

### The embryonic lethality of *mdmx* knockout mice is fully rescued by *p53*^*3KR/3KR*^

**Figure 1:**
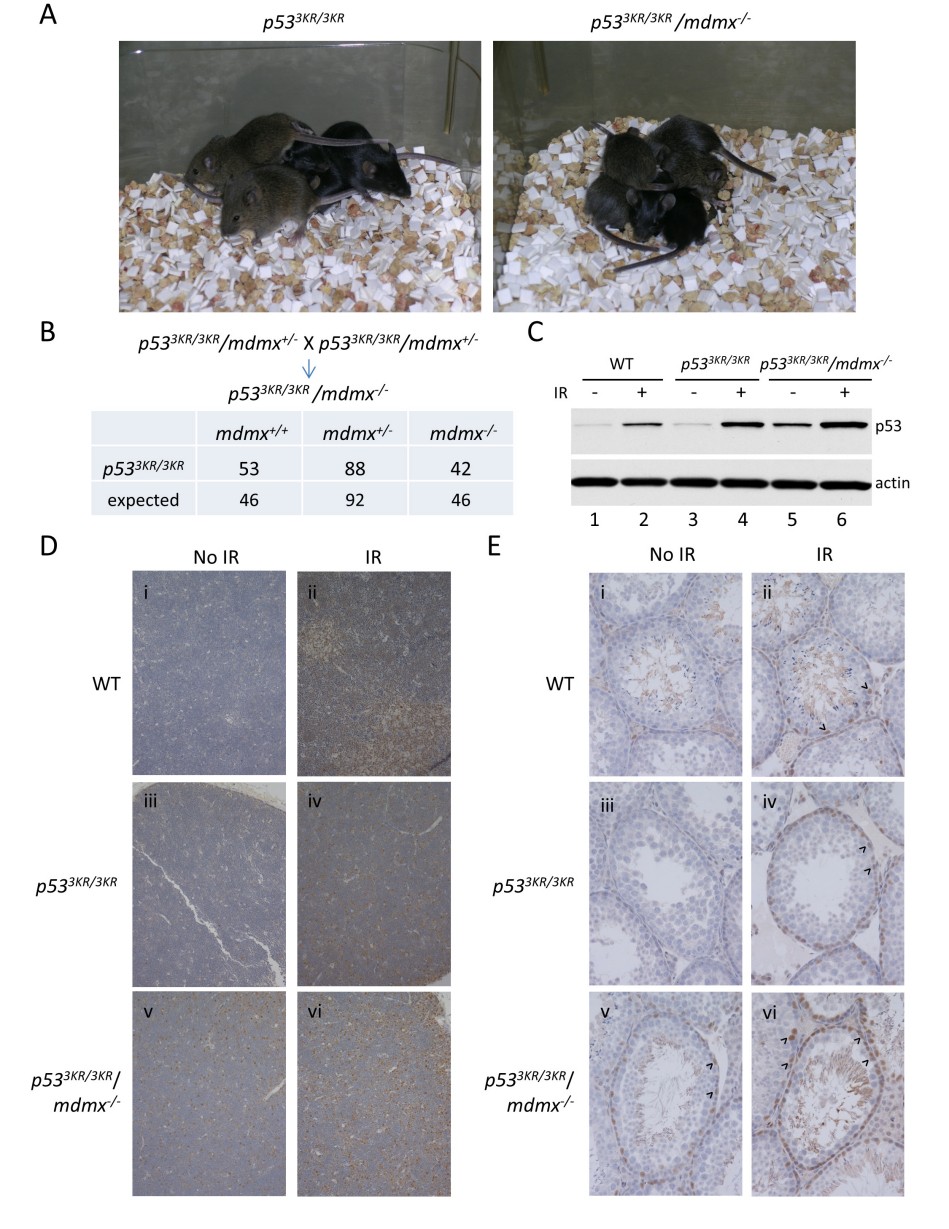
The embryonic lethality of *mdmx* knockout is rescued by *p53 3KR* mutation, and p53 3KR protein has increased stability in the absence of Mdmx **A.** The representative pictures of *p53*^*3KR/3KR*^ and *p53*^*3KR/3KR*^*/mdmx*^*-/-*^ mice at weaning. **B.** Intercross of *p53*^*3KR/3KR*^*/mdmx*^*+/-*^ mice. The numbers of the resulting progeny are listed in the table. The expected numbers of progeny of different genotypes are based on the total number of progeny from the cross. **C.** Western Blotting of the protein extracts of thymi from wild-type, *p53*^*3KR/3KR*^, and *p53*^*3KR/3KR*^*/mdmx*^*-/-*^ mice treated without (lanes 1, 3, and 5) or with (lanes 2, 4, and 6) ionizing radiation. **D.** Immunohistochemical staining of p53 in thymus from i and ii, wild-type; iii and iv, *p53*^*3KR/3KR*^; v and vi, *p53*^*3KR/3KR*^*/mdmx*^*-/-*^ mice treated without (i, ii, iii) or with (ii, iv, vi) radiation. **E.** Immunohistochemical staining of p53 in testis from 2-month-old i and ii, wild-type; iii and iv, *p53*^*3KR/3KR*^; v and vi, *p53*^*3KR/3KR*^*/mdmx*^*-/-*^ mice treated without (i, ii, iii) or with (ii, iv, vi) ionizing radiation.

It is established that the lethality of *mdmx* knockout mice is caused by p53-mediated growth arrest [19]; in addition, the lethality of *mdmx* knockout mice is rescued by a mutant *p53* with reduced transcriptional signaling in cell cycle arrest and apoptotic responses [20]. To determine if the p53 3KR mutant, an acetylation-defective mutant which is unable to induce senescence and cell-cycle arrest [9], can rescue the embryonic lethality of *mdmx* mutant mice, *p53*^*3KR/3KR*^ mice were crossed with *mdmx* heterozygote knockout mice. We first generated mice homozygous for the *p53 3KR* mutant allele and heterozygous for the *mdmx* knockout allele (*p53*^*3KR/3KR*^*/mdmx*^*+/-*^ mice). Intercrosses of *p53*^*3KR/3KR*^*/mdmx*^*+/-*^ mice is expected to yield 25% of offspring to be *p53*^*3KR/3KR*^*/mdmx*^*-/-*^ mice if embryonic lethality of *mdmx* knockout mice can be rescued by *p53 3KR* mutant. From nearly 200 live mice generated from the cross, about a quarter of them were genotyped to be *p53*^*3KR/3KR*^*/mdmx*^*-/-*^ mice, close to Medelian ratio (Figure 1B). Moreover, *p53*^*3KR/3KR*^*/mdmx*^*-/-*^ mice were apparently normal without developmental defects (Figure 1A). These data demonstrated that the embryonic lethality of *mdmx* knockout mice was fully rescued by the *p53 3KR* mutation, providing direct evidence that the lethality of *mdmx* knockout mice is caused by p53-dependent cell growth suppression [19].

### Increased stability of p53 3KR protein in the absence of Mdmx

Despite its high sequence homology with Mdm2 and the presence of a RING domain, Mdmx does not have intrinsic E3-ligase activity for p53 [24]. Although several studies indicate that the Mdm2/Mdmx complex is critical for p53 degradation [25, 26], it remains unclear whether Mdmx is absolutely required for this event *in vivo*. To address this question, we set out to investigate whether the abundance of p53 3KR protein was affected in the absence of Mdmx. Briefly, wild-type mice, *p53*^*3KR/3KR*^, and *p53*^*3KR/3KR*^*/mdmx*^*-/-*^ mice were given ionizing radiation to induce DNA damage *in vivo*, after which tissues were collected and analyzed by western blot and immunohistochemistry. Briefly, whole cell extracts were prepared from thymi of wild-type, *p53*^*3KR/3KR*^, and *p53*^*3KR/3KR*^*/mdmx*^*-/-*^ mice with or without ionizing radiation and the protein levels of p53 were determined by Western blot. There was no obvious difference in p53 levels in *p53*^*3KR/3KR*^ mice compared to wild-type p53 mice without radiation (Figure 1C lane 3 vs. 1), whereas in *p53*^*3KR/3KR*^*/mdmx*^*-/-*^ mice, the levels of p53 3KR protein was slightly higher than that of wild-type and *p53*^*3KR/3KR*^ mice in the absence of DNA damage (Figure 1C lane 5 vs. 1 and 3). Notably, in respond to ionizing radiation, the levels of p53 were increased in mice of all genotypes (Figure 1C lanes 2, 4, and 6, vs. 1, 3, 5, respectively), indicating a normal response to DNA damage in *p53*^*3KR/3KR*^*/mdmx*^*-/-*^ mice, compared to wild type and *p53*^*3KR/3KR*^ mice.

To further demonstrate the effect of *mdmx* deletion on p53 stability at the cellular level, the p53 abundance was visualized by immunostaining using anti-p53 antibody. In the thymus from *p53*^*3KR/3KR*^ mice, there was no obvious increase of p53 levels in the absence of radiation, compared to the thymus from wild-type mice, shown by the immunohistochemical staining of p53 (Figure 1D iii vs i). In contrast, there were modestly increases of p53 protein levels in thymus from *p53*^*3KR/3KR*^*/mdmx*^*-/-*^ mice compared to the ones from p53 wild-type and *p53*^*3KR/3KR*^ mice (Figure 1D v vs i and iii). However, after ionizing radiation, p53 was further induced in mice of all genotypes in response to DNA damage (Figure 1D ii, iv, and vi vs. i, iii, and v, respectively).

Similar results were also obtained in testis which showed restricted p53 staining mostly in primary spermatogonia. There were more primary spermatogonia with p53 staining in testis from *p53*^*3KR/3KR*^*/mdmx*^*-/-*^ mice, compared to wild type and *p53*^*3KR/3KR*^ mice (Figure 1E iii vs. i and ii). The number of p53 positive cells was further increased after ionizing radiation (Figure 1E ii, iv, and vi vs. i, iii, and v, respectively). Increase of p53 abundance was also observed in other tissues including spleen, pancreas, small intestine, cerebral cortex, hippocampus in *p53*^*3KR/3KR*^*/mdmx*^*-/-*^ mice, compared to wild type and *p53*^*3KR/3KR*^ mice (Supplementary Figure 1 7 vs. 1 and 4; 8 vs. 2 and 5; 9 vs 3 and 6; 16 vs. 10 and 13; 17 vs 11 and 14). Consistent with the previous study [27], the protein levels of p53 remained low in liver in *p53*^*3KR/3KR*^*/mdmx*^*-/-*^ mice, similar to that in wild-type and *p53*^*3KR/3KR*^ mice (Supplementary Figure 1 18 vs. 12 and 15), even after ionizing radiation (data not shown), suggesting that the role of Mdmx in p53 degradation is potentially tissue-specific. Notably, as expected, DNA damage induced activation of caspase3, occurred only in wild-type mice after ionizing radiation, but not in *p53*^*3KR/3KR*^ or *p53*^*3KR/3KR*^*/mdmx*^*-/-*^ mice, demonstrating the inability to induce apoptotic response by p53 3KR mutant in the absence of Mdmx (Supplementary Figure 2A ii vs. iv and vi; S2B ii vs. iv and vi). Taken together, these results provide compelling evidences suggesting that Mdmx is only partially required for p53 degradation *in vivo*. Moreover, Mdmx-mediated effects on p53 degradation are apparently modest and tissue-specific.

### Improved metabolism in *p53*^*3KR/3KR*^*/mdmx*^*-/-*^ mice

**Figure 2:**
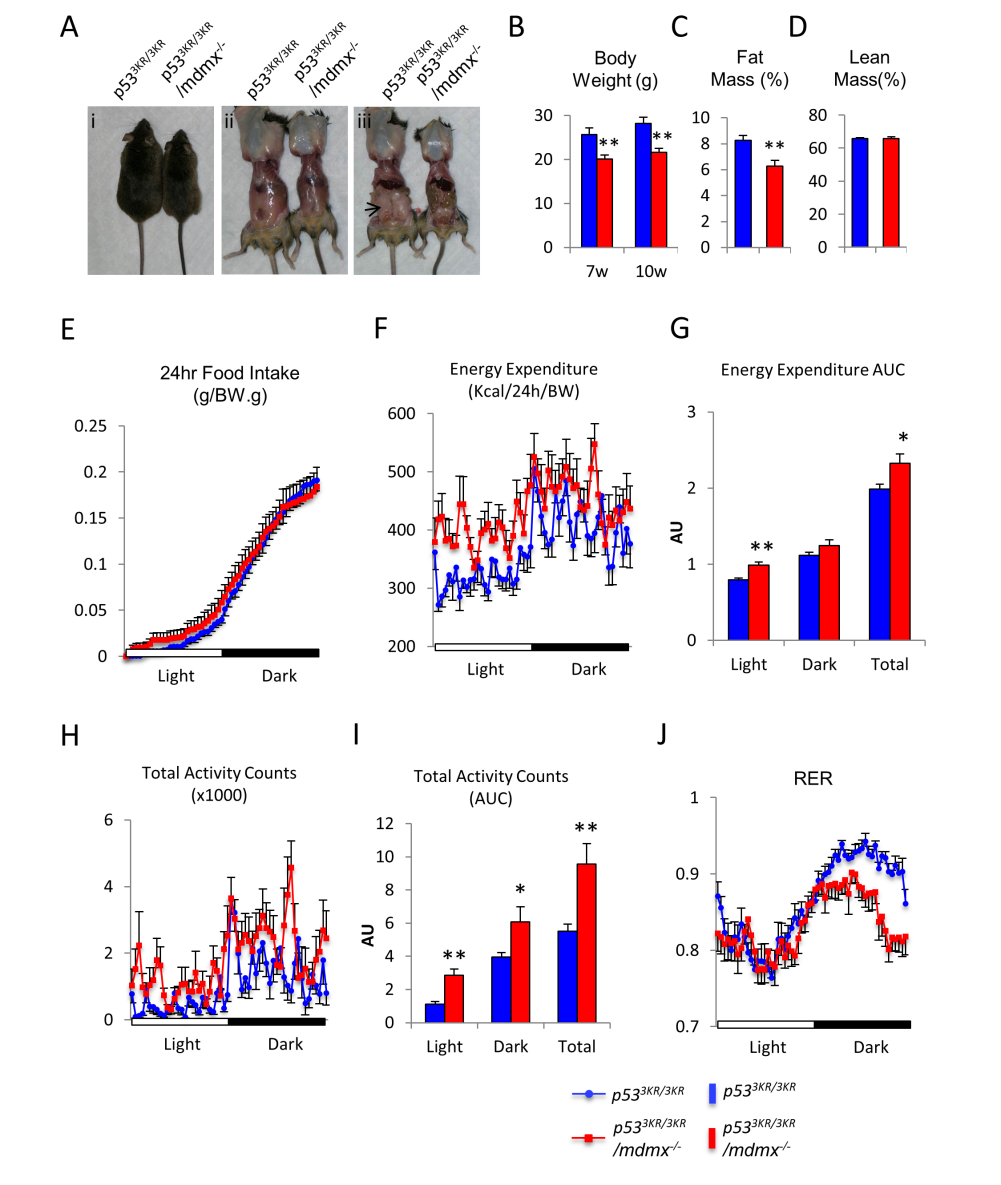
*p53*^*3KR/3KR*^*/mdmx*^-/-^ mice have reduced body fat **A.** Gross appearances of 4-month-old *p53*^*3KR/3KR*^ and *p53*^*3KR/3KR*^*/mdmx*^*-/-*^ mice and dissection to reveal body fat. Arrow in iii indicates the prominent epididymal fat in *p53*^*3KR/3KR*^ mice. **B.** Comparisons of body weight. **C.** Measurements of fat mass content and **D.** Measurements of lean mass content in male mice at 10 weeks of age (*n* = 7, 5). **E.**-**J.** Calorimetric analyses of *p53*^*3KR/3KR*^*/mdmx*^*-/-*^ mice (*n* = 8) and *p53*^*3KR/3KR*^ mice (*n* = 8). **E**. Normalized food intake during a period of 24 hours. **F**. Recording of the energy expenditure during a period of 24 hours. **G**. Area under curve (AUC) of energy expenditure. **H**. Recording of total activity counts during a period of 24 hours. **I**. Area under curve (AUC) of total activity counts. **J**. Recording of respiratory exchange ratio (RER) during a period of 24 hours. * represents *p* < 0.05, ** represents *p* < 0.01. Data are represented as mean ± SEM.

*p53*^*3KR/3KR*^*/mdmx*^*-/-*^ mice had normal life span similar to that of *p53*^*3KR/3KR*^ mice, and they also did not show early onset of spontaneous tumor. However, adult *p53*^*3KR/3KR*^*/mdmx*^*-/-*^ mice appeared to be skinnier than *p53*^*3KR/3KR*^ control mice at 4 months of age (Figure 2Ai). Since our previous study showed that there were no significant body weight differences between wild type and *p53*^*3KR/3KR*^ mice (Supplementary Figure 3A) [28], and there were no differences in metabolic activities such as glucose tolerance test between wild type and *p53*^*3KR/3KR*^ mice (Supplementary Figure 3B), the skinny phenotypes observed in *p53*^*3KR/3KR*^*/mdmx*^*-/-*^ mice were most likely caused by loss of Mdmx. Anatomical analysis revealed a gross reduction of fat mass, most noticeably epididymal fat, in *p53*^*3KR/3KR*^*/mdmx*^*-/-*^ mice (Figure 2Aii and iii). The body weight of *p53*^*3KR/3KR*^*/mdmx*^*-/-*^ mice measured at 7-week and 10-week old were about 20% lower than their littermate controls (Figure 2B). To reveal the cause for the differences in body weight, Magnetic Resonance Imaging (MRI) was performed on these mice. Consistent to the skinny phenotype, the MRI results showed that there was a significant reduction of the fat content in *p53*^*3KR/3KR*^*/mdmx*^*-/-*^ mice compared to *p53*^*3KR/3KR*^ mice (Figure 2C). In contrast, there was a similar lean body mass between these mice (Figure 2D).

The lean body phenotype in *p53*^*3KR/3KR*^*/mdmx*^*-/-*^ mice implied potentially different metabolic activities compared to the control mice. Since body weight is solely determined by the balance between food intake and energy expenditure, calorimetric analysis was performed to monitor food intake, locomotor activities, O_2_ consumption and CO_2_ production to reveal the underlying metabolic changes in these mice [29]. Age matched *p53*^*3KR/3KR*^*/mdmx*^*-/-*^ and *p53*^*3KR/3KR*^ mice were housed individually in enclosed metabolic cages with continues recording. Despite the differences in body weight, the results showed that normalized food intake based on their body weight was comparable between *p53*^*3KR/3KR*^*/mdmx*^*-/-*^ and the control mice (Figure 2E), suggesting that their lean phenotype was not caused by reduced energy intake but by increases of energy usage. Consistently, the biometric analysis showed that *p53*^*3KR/3KR*^*/mdmx*^*-/-*^ mice had significantly higher energy expenditure (Figure 2F and 2G), which was also supported by increased locomotor activity compared to *p53*^*3KR/3KR*^ controls (Figure 2H and 2I). Moreover, the biometric analysis also measures release of CO_2_ and consumption of O_2_ from each mouse to determine the respiratory exchange ratio (RER, CO_2_ released over O_2_ consumed). Since more oxygen is required to fully oxidize fatty acids than carbohydrates (RER value for 100% utilization of carbohydrates is 1, whereas 100% utilization of fatty acids is 0.75, which means the lower the RER is, the more fatty acid is utilized), differences in RER reflect the preferential usage of fatty acids or carbohydrates in metabolic activities. Significantly, the RER was lower for *p53*^*3KR/3KR*^*/mdmx*^*-/-*^ mice than the control mice (Figure 2J), indicating a preference of utilizing fatty acids over carbohydrates in *p53*^*3KR/3KR*^*/mdmx*^*-/-*^ mice compared to *p53*^*3KR/3KR*^ mice, which subsequently provides more energy to support their activities in *p53*^*3KR/3KR*^*/mdmx*^*-/-*^ mice. Taken together, these data suggest that the lean phenotype in *p53*^*3KR/3KR*^*/mdmx*^*-/-*^ mice was caused by increased energy expenditure through preferential usage of fatty acids as fuel, which generates more energy than carbohydrates and other nutrients.

### *p53*^*3KR/3KR*^*/mdmx*^*-/-*^ mice are protected against obesity and the comorbidities

**Figure 3:**
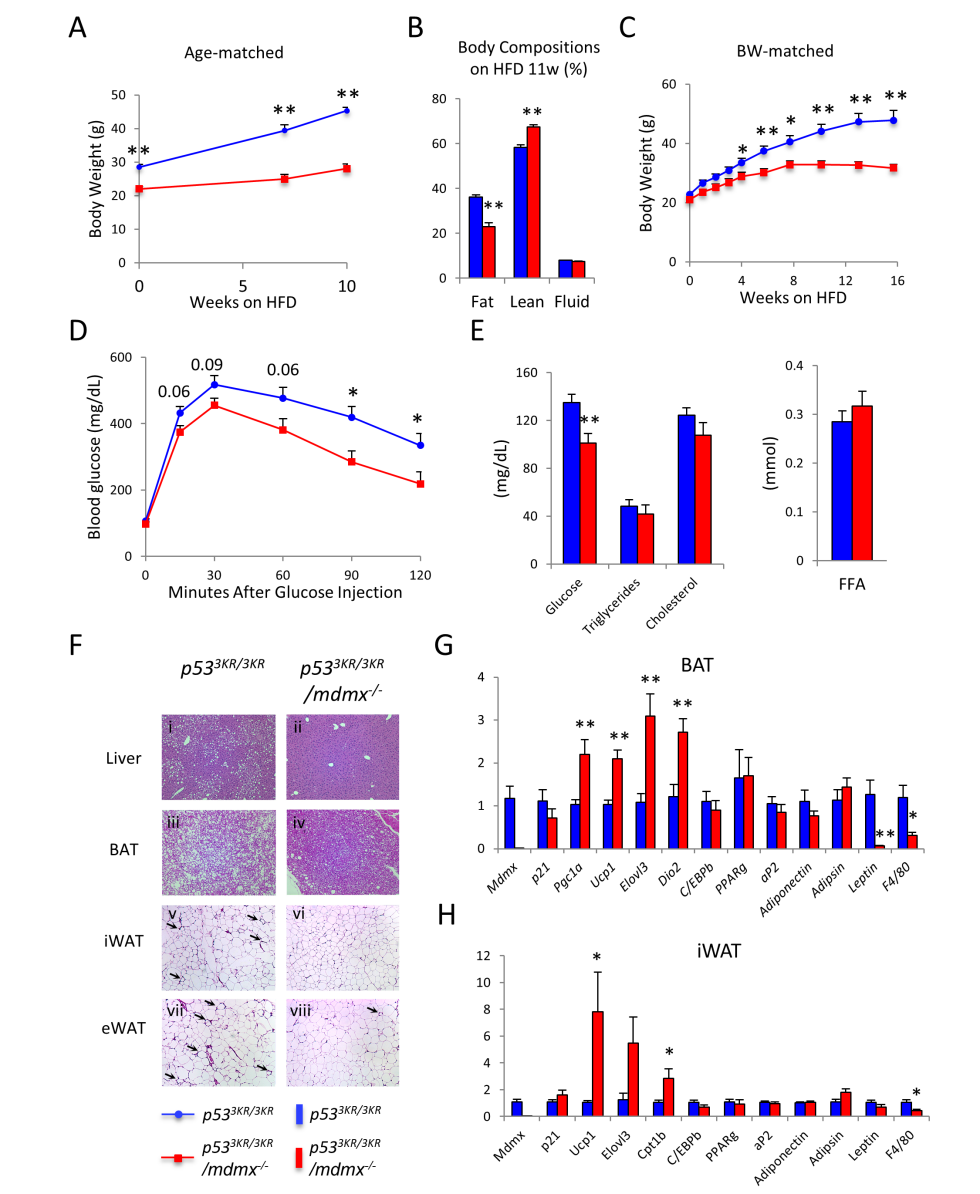
*p53*^*3KR/3KR*^*/mdmx*^-/-^ mice resist diet-induced obesity **A.** Body weight curve of age-matched *p53*^*3KR/3KR*^ (*n* = 11) and *p53*^*3KR/3KR*^*/mdmx*^*-/-*^ mice (*n* = 8) on high fat diet. **B.** Body composition of mice used in (A) after 10 weeks on HFD. **C.** Average body weight curve of weight-matched *p53*^*3KR/3KR*^ and *p53*^*3KR/3KR*^*/mdmx*^*-/-*^ mice on HFD (*n* = 5, 5). **D.** Glucose tolerance test on mice after 10 weeks on HFD. **E.** Plasma levels of glucose, triglycerides, cholesterol, and free fatty acids in mice after 12 weeks HFD feeding. Mice were sacrificed after overnight fasting (*n* = 6, 6). **F.** Histopathological analysis of *p53*^*3KR/3KR*^ and *p53*^*3KR/3KR*^*/mdmx*^*-/-*^ mice on HFD. *p53*^*3KR/3KR*^ (i, iii, v, and vii) and *p53*^*3KR/3KR*^*/mdmx*^*-/-*^ (ii, iv, vi, and viii) mice. i and ii, liver; iii and iv, brown adipose tissue (BAT); v and vi, subcutaneous inguinal white adipose tissue (iWAT); vii and viii, visceral epididymal white adipose tissue (eWAT). Arrows indicate the “crown” like structures associated with macrophage infiltration. **G.** Relative expression levels of genes of interest in BAT (*n* = 6, 6). **H.** Relative expression levels of genes of interest in iWAT (n = 6, 6). *p < 0.05, **p < 0.01. Data are represented as mean ± SEM.

Given their lean phenotypes, we asked whether *p53*^*3KR/3KR*^*/mdmx*^*-/-*^ mice have metabolic benefits, particularly in protection against obesity. To address this question, *p53*^*3KR/3KR*^*/mdmx*^*-/-*^ and the control *p53*^*3KR/3KR*^ mice were fed with high fat diet (HFD, 60% fat calorie composition) to induce obesity [30]. Consistent to their lean phenotypes on regular chow diet, *p53*^*3KR/3KR*^*/mdmx*^*-/-*^ mice gained much less weight during HFD feeding compared to *p53*^*3KR/3KR*^ mice on high fat diet, resulting in a significant protection from obesity (Figure 3A). *p53*^*3KR/3KR*^*/mdmx*^*-/-*^ mice showed a nearly 40% less of body fat content, 21% fat content in *p53*^*3KR/3KR*^*/mdmx*^*-/-*^ mice compared to 38% fat content in *p53*^*3KR/3KR*^ mice, while the percentage of the lean mass was increased in *p53*^*3KR/3KR*^*/mdmx*^*-/-*^ mice as determined by MRI (Figure 3B). To exclude the effects of initial weight differences between *p53*^*3KR/3KR*^*/mdmx*^*-/-*^ and *p53*^*3KR/3KR*^ mice on the outcome of diet-induced obesity, bodyweight-matched *p53*^*3KR/3KR*^*/mdmx*^*-/-*^ and *p53*^*3KR/3KR*^ mice were treated with HFD. Again, protection against obesity in *p53*^*3KR/3KR*^*/mdmx*^*-/-*^ mice was recapitulated (Figure 3C).

Obesity often leads to a series of co-morbidities, primarily insulin resistance and type 2 diabetes [31, 32]. Insulin resistance and type 2 diabetes can be assessed by glucose tolerance test to determine the capability of mice to maintain glucose homeostasis in response to blood glucose shock. In short, a bolus of glucose was injected intraperitoneally into the mice and the levels of blood glucose were determined. Compared to *p53*^*3KR/3KR*^ control mice, *p53*^*3KR/3KR*^*/mdmx*^*-/-*^ mice showed lower glucose levels after 1-hour post glucose injection (Figure 3D), indicating a more efficient glucose clearance. This improved glucose homeostasis in *p53*^*3KR/3KR*^*/mdmx*^*-/-*^ mice were further supported by their lower *ad libitum* blood glucose levels than those of *p53*^*3KR/3KR*^ mice (Figure 3E).

Obesity also causes nonalcoholic fatty liver diseases (NAFLD), a risk factor for cirrhosis and hepatocellular carcinoma [33-35]. After high fat diet treatment, there was apparent accumulation of lipid droplets in the liver (hepatic steatosis) from *p53*^*3KR/3KR*^ mice from the histopathological analysis, in contrast, the hepatic steatosis in *p53*^*3KR/3KR*^*/mdmx*^*-/-*^ mice was largely prevented indicated by significantly reduced lipid droplets (Figure 3F ii vs. i). Interestingly, there were no significant differences in the expression of genes regulating lipogenesis, gluconeogenesis, lipid oxidation or ketogenesis in the liver (Supplementary Figure 4A). Moreover, the circulating triglycerides, cholesterol, and free fatty acids (FFA) levels were unchanged (Figure 3E), suggesting that the anti-obesity effects in *p53*^*3KR/3KR*^*/mdmx*^*-/-*^ mice, were not originated in liver.

### Improved adipose catabolism in *p53*^*3KR/3KR*^*/mdmx*^*-/-*^ mice

Since the lean phenotype in *p53*^*3KR/3KR*^*/mdmx*^*-/-*^ mice is not caused by reduced food intake (Figure 2A), it is unlikely that the metabolic improvements in *p53*^*3KR/3KR*^*/mdmx*^*-/-*^ mice were stemmed from the central nervous system. Furthermore there were minimal changes in liver metabolism (Supplementary Figure 4A), suggesting other peripheral metabolic tissues played significant roles in the skewed metabolic activities in *p53*^*3KR/3KR*^*/mdmx*^*-/-*^ mice.

Given that dramatic reduction of body fat composition and increased energy expenditure accompanied by preference of lipid utilization in *p53*^*3KR/3KR*^*/mdmx*^*-/-*^ mice, we reasoned that the anti-obesity effect in these mice potentially arose from either reduced lipid production or increased lipid catabolism in adipose tissues. To test these hypotheses, major fat tissues from HFD treated mice were collected and subjected to histopathological and gene expression analyses.

There are two types of adipose tissues in mice, brown adipose tissue (BAT) and white adipose tissue (WAT). BAT has characteristic high levels of expression of UCP1 due to its function to produce heat. WAT, which stores excess energy, can be further divided into subcutaneous WAT (under the skin WAT, including subcutaneous inguinal WAT (iWAT)) and visceral WAT (abdominal WAT, including epididymal WAT (eWAT)). We first looked at the BAT since it plays a major role in consuming lipids to generate heat. The results of histology showed that there was reduced lipid accumulation in brown adipose tissue (BAT) in *p53*^*3KR/3KR*^*/mdmx*^*-/-*^ mice (Figure 3F iv vs. iii). Furthermore, the expression of genes *Ucp1, ELOVL3, Dio2* and *Pgc-1α* were up-regulated. There genes are normally associated with lipid oxidation, indicating an increased usage of lipid in the BAT of *p53*^*3KR/3KR*^/*mdmx*^*-/-*^ mice, compared to the control mice (Figure 3G). In contrast, the white adipocyte-specific gene *Leptin* was significantly down-regulated, whereas the pan-adipocyte genes, including *Pparγ, aP2, Adiponectin, Adipsin* and *C/ebpβ*, were not affected in the BAT of *p53*^*3KR/3KR*^/*mdmx*^*-/-*^ mice, suggesting similar adipocyte development between theses mice (Figure 3G).

In white adipose tissues (WAT), the size of the adipocytes were also smaller in *p53*^*3KR/3KR*^*/mdmx*^*-/-*^ mice than in *p53*^*3KR/3KR*^ control mice (Figure 3F iv vs. v, iWAT: subcutaneous inguinal white adipose tissue; viii vs. vii, eWAT: visceral epididymal white adipose tissue), indicating reduced storage of lipid in WAT in *p53*^*3KR/3KR*^*/mdmx*^*-/-*^ mice on HFD. Strikingly, there was a similar up-regulation of brown adipocyte-related genes, *Ucp1, ELOVL3* and *Cpt-1b* while the pan-adipocyte gene expression remained unchanged in the iWAT from *p53*^*3KR/3KR*^*/mdmx*^*-/-*^ mice (Figure 3H), indicating a selective expression of brown adipocyte-related genes in *p53*^*3KR/3KR*^/*mdmx*^*-/-*^ mice. The higher transcriptional activities of *Ucp1, ELOVL3* and *Cpt-1b* in iWAT, which are typically associated with “browning” in WAT, a process to increase output of heat in response to cold environment, were reminiscent of the similar changes in BAT in *p53*^*3KR/3KR*^/*mdmx*^*-/-*^ mice compared to the control mice, further indicating an increase of lipid usage. In addition, visceral epididymal WAT (eWAT) is normally dormant in metabolic activities and its accumulation leads to higher risks of metabolic diseases [36, 37]. The expressions of BAT associated genes in the eWAT were much lower compared to that in the iWAT and BAT, and they were not affected by the ablation of mdmx (Supplementary Figure 4B). Interestingly, the key lipid oxidative genes Cpt-1a and Acox1 were up-regulated, buttressing their smaller adipocyte size in *p53*^*3KR/3KR*^*/mdmx*^*-/-*^ mice.

Obesity causes macrophage infiltration in fat and chronic inflammation, which in turn contributes to insulin resistance [38-41]. In addition, previous study also demonstrated the role of p53 in macrophage functions [42], prompted us to determine macrophage related phenotypes. Less “crown” structures were observed, the sign of macrophage infiltration, in iWAT eWAT (Figure 3F vi vs. v; viii vs. vii), which was further supported by lower levels of macrophage marker F4/80 in BAT, both in iWAT and eWAT in *p53*^*3KR/3KR*^*/mdmx*^*-/-*^ mice than in *p53*^*3KR/3KR*^ mice (Figure 3G, 3H, and Supplementary Figure 4B), indicating a better adipose health with lower inflammation in *p53*^*3KR/3KR*^*/mdmx*^*-/-*^ mice than in *p53*^*3KR/3KR*^ mice. Taken together, these data suggest BAT associated genes including lipid oxidative genes were selectively activated in fat tissues in *p53*^*3KR/3KR*^*/mdmx*^*-/-*^ mice, resulting in increased energy production and fat dissipation, leading to protection against diet-induced obesity and prevention of insulin resistance in *p53*^*3KR/3KR*^*/mdmx*^*-/-*^ mice.

### Brown remodeling of WAT in *p53*^*3KR/3KR*^*/mdmx*^*-/-*^ mice

**Figure 4:**
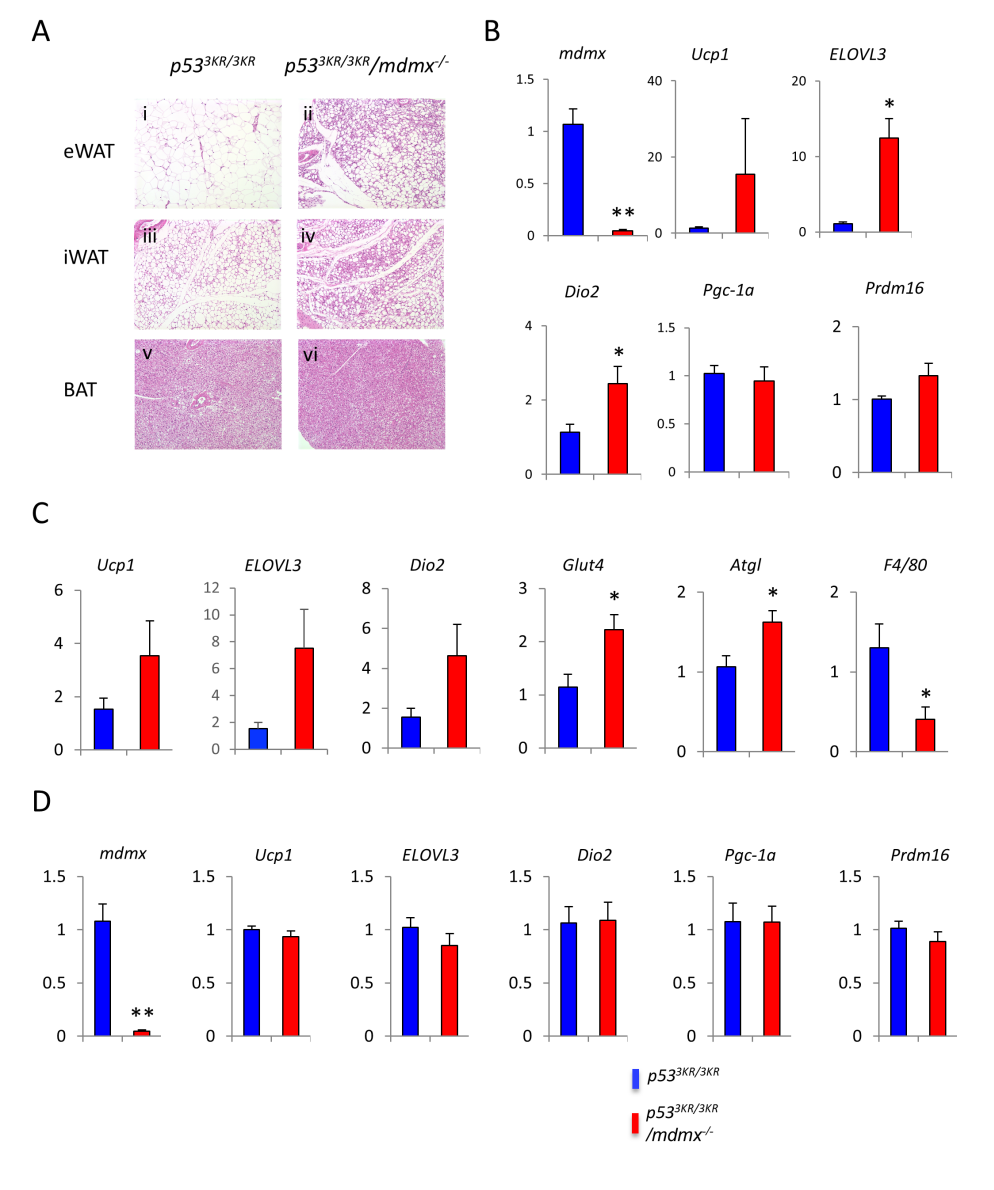
Increased thermogenic response in *p53*^*3KR/3KR*^*/mdmx*^-/-^ mice 4-month old male mice were exposed to 4 °C for 4 days. **A.** Histology of adipose tissues from *p53*^*3KR/3KR*^ (i, iii, and v) and *p53*^*3KR/3KR*^*/mdmx*^*-/-*^ (ii, iv, and vi) mice on high fat diet. **B.** Relative expression levels of genes of interest in eWAT. **C.** Relative expression levels of genes of interest in iWAT. **D**. Relative expression levels of genes of interest in BAT. *p < 0.05, **p < 0.01 (*n* = 7, 5). Data are represented as mean ± SEM.

To further demonstrate the anti-obesity effects through up-regulation of BAT associated genes particularly in WAT, *p53*^*3KR/3KR*^*/mdmx*^*-/-*^ and control mice were exposed to cold environment, an established method to activate browning (non-shivering thermogenesis) in white adipose tissues. After 4 days in the cold, the subcutaneous inguinal WAT (iWAT) and visceral epididymal WAT (eWAT) from *p53*^*3KR/3KR*^*/mdmx*^*-/-*^ mice showed marked morphological differences, compared to those from *p53*^*3KR/3KR*^ mice. Typically, adipocytes in WAT are unilocular cells fulfilled with a large lipid droplet (Figure 4Ai). After cold exposure, multilocular brown-like adipocytes emerge in iWAT (Figure 4Aiii), a process variously referred to as “browning”, “beiging”, or “brown remodeling”. The prevalence of the multilocular adipocytes in the browning-prone iWAT was much higher in *p53*^*3KR/3KR*^*/Mdmx*^*-/-*^ than in the control mice (Figure 4A iv vs. iii). In contrast to iWAT, eWAT are normally resistant to browning, even after cold challenge, indicated by the maintaining of the unilocular cell morphology (Figure 4Ai). Surprisingly, a significant number of multilocular adipocytes appeared in eWAT in *p53*^*3KR/3KR*^*/mdmx*^*-/-*^ mice (Figure 4Aii), indicating an expansion of browning in *p53*^*3KR/3KR*^*/mdmx*^*-/-*^ mice compared to *p53*^*3KR/3KR*^ mice. Consistent to the presence of browning in eWAT, the expression of the representative brown adipocyte markers Ucp1, *ELOVL3* and *Dio2* were increased significantly in eWAT in *p53*^*3KR/3KR*^*/mdmx*^*-/-*^ mice (Figure 4B). In iWAT, higher expression of *Ucp1*, *ELOVL3* and *Dio2* was observed in *p53*^*3KR/3KR*^*/mdmx*^*-/-*^ mice compared to *p53*^*3KR/3KR*^ mice, however, the increases were not statistically significant probably due to increased expression of these same genes in iWAT from *p53*^*3KR/3KR*^ mice and the large variations among the samples (Figure 4C). Nonetheless, the expression of Glut4, an insulin-dependent glucose transporter, increased over two-fold in *p53*^*3KR/3KR*^*/mdmx*^*-/-*^ mice, so did the major adipocyte lipase Atgl, suggesting that increase of glucose uptake and lipolysis to provide fuels to sustain the elevated metabolic activities. Consistent with the phenotypes of mice treated with HFD, inflammation in adipose tissues in *p53*^*3KR/3KR*^*/mdmx*^*-/-*^ mice remained low, supported by the consistently low levels of the macrophage marker F4/80 (Figure 4C). In BAT, the thermogenic program was maximally activated in both *p53*^*3KR/3KR*^*/mdmx*^*-/-*^ mice and *p53*^*3KR/3KR*^ mice during cold challenge, and differences were minimal between these mice (Figure 4A vi vs. v, and Figure 4D). Taken together, these results further supported that there was an increase of browning of WAT in *p53*^*3KR/3KR*^*/mdmx*^*-/-*^ mice, consistent with the increase of lipid usage and the anti-obesity effect during HFD feeding in these mice.

### ELOVL3 is a novel target of p53

**Figure 5:**
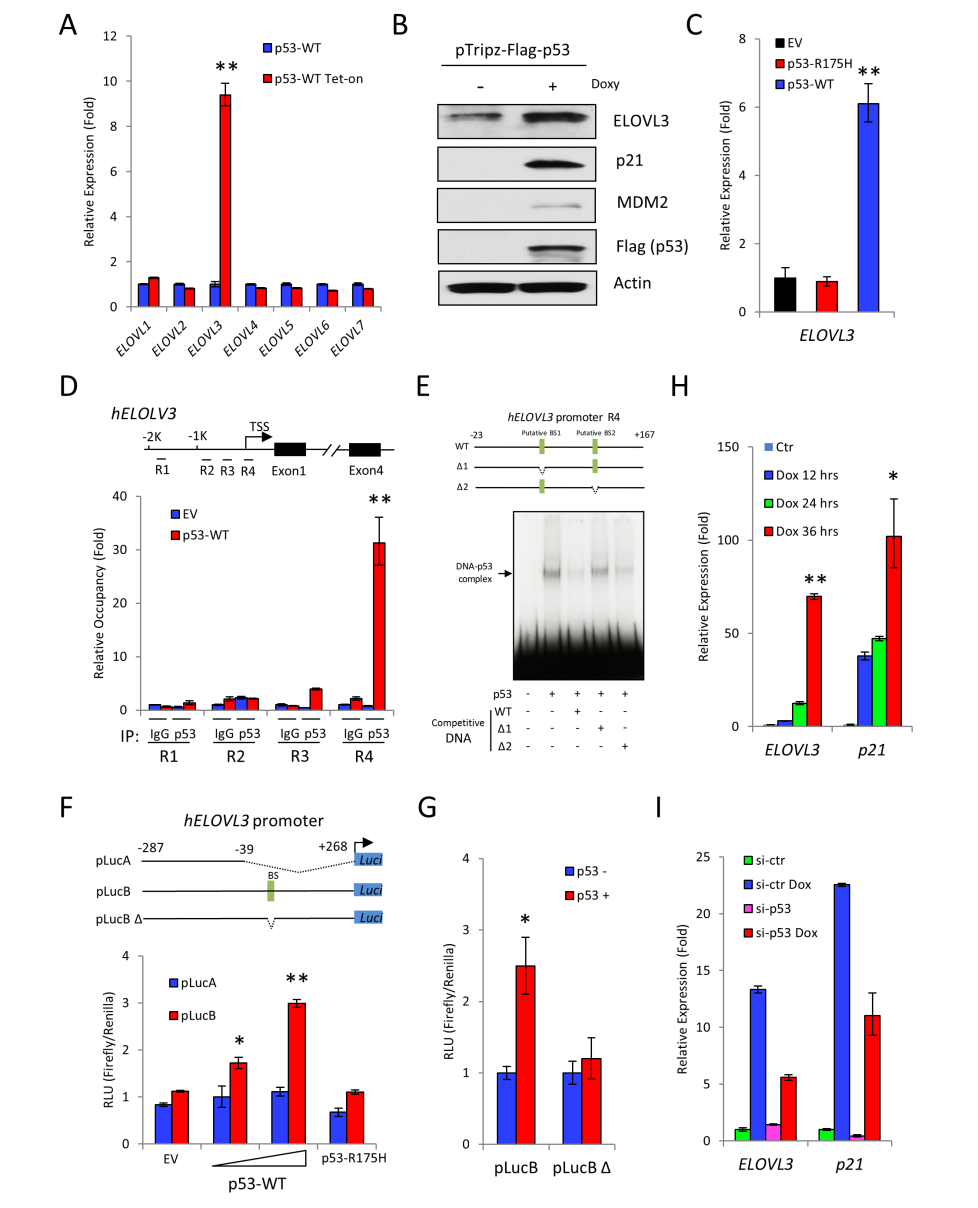
ELOVL3 is a transcriptional target of p53 **A.** The p53 dependent expression of *ELOVL* family genes was measured by qPCR. The expression of *p53* was induced by doxycycline in inducible H1299 p53 stable cell line. **B.** Western blot to determine the levels of ELOVL3 and p53 target genes p21 and MDM2 expression before and after induced *p53* expression by doxycycline in inducible H1299 p53 stable cell line. **C.** Relative expression levels of *ELOVL3* were determined by qPCR after transient expression of wild-type *p53* or mutant p53 R175H. Transfection with empty vector was used as control. **D.** Regions of *ELOVL3* promoter were tested for p53 binding by qChIP using control IgG and anti-p53 antibody. **E.** Electro mobility shift assay was performed by using probes containing full length or partially deleted *ELOVL3* promoter. **F.** Luciferase assay using full length ELOVL3 promoter (pLucB) or partially deleted ELOVL3 promoter (pLucA) after transfection of increasing levels of p53, or mutant p53-R175H for 24 hours. **G.** Luciferase assay using luciferase expression vector with full length (pLucB) or partially deleted (pLucB Δ) *ELOVL3* promoter in the absence or presence of *p53* expression. **H.** Relative levels of *ELOVL3* upon DNA damage determined by qPCR after H460 cells were treated with 1 uM doxorubicin for 12, 24 or 36 hrs. **I**. Control or p53 knockdown H460 cells were treated with or without 1 uM doxorubicin for 24 hrs. The expression of *ELOVL3* and p53 target gene *p21* was determined by qPCR. * represents *p* < 0.05;** represents *p* < 0.01.

Since the levels of p53 3KR protein were increased in *p53*^*3KR/3KR*^*/mdmx*^*-/-*^ mice compared to *p53*^*3KR/3KR*^ mice, we reasoned that there would be an increase of transcription to support the browning in *p53*^*3KR/3KR*^*/mdmx*^*-/-*^ mice. It has been demonstrated that the expression of ELOVL3 is closely associated with BAT and its metabolic activities [43-45]. Because the increased expression of *ELOVL3* was consistently observed in *p53*^*3KR/3KR*^*/mdmx*^*-/-*^ mice compared to *p53*^*3KR/3KR*^ mice, we sought to investigate whether *ELOVL3* is a bona fide transcriptional target of p53, which serves as a potential mediator for the increased brown remodeling in *p53*^*3KR/3KR*^*/mdmx*^*-/-*^ mice. *ELOVL* family members are fatty acid elongases that catalyze fatty acid elongation [46, 47]. Among the seven known *ELOVL* family members, only *ELOVL3* expression levels was increased after p53 induction, as determined by qPCR (Figure 5A). Furthermore, ELOVL3 protein levels also increased following expression of p53 in H1299 cells. As expected, the levels of p53 target genes p21 and mdm2 were also increased under the same condition, suggesting activation of p53 dependent transcription (Figure 5B). The induction of *ELOVL3* is dependent on the integrity of DNA binding domain of p53, since p53 DNA binding deficient mutant, p53-R175H failed to increase ELOVL3 expression levels, whereas wild-type p53 did (Figure 5C). To identify the p53 binding sites in the *ELOVL3* promoter, regions of *ELOVL3* promoter were tested in quantitive chromatin immumoprecipitation (qChIP) assay. Only region 4 as depicted in the diagram displayed significant recruitment of p53 protein (Figure 5D). Upon further analysis, there are two putative p53 binding sites within region 4 of *ELOVL3* promoter, which prompted us to narrow down the p53 binding site. As shown in the competition gel-shift assay, the binding between p53 and ^32^P-labelled wild type *ELOVL3* promoter DNA probe was reduced by unlabeled *ELOVL3* promoter DNA containing deletion of binding site 2 (BS2), but not by *ELOVL3* promoter DNA containing deletion of binding site 1 (BS1), indicating BS1 is the binding site for p53 on *ELOLV3* promoter (Figure 5E). To validate the transcriptional regulation of *ELOVL3* by p53, luciferase expression plasmid containing wild type *ELOVL3* promoter (pLucB), or containing *ELOVL3* promoter with large deletion (pLucA), or containing *ELOVL3* promoter with deletion of the p53 binding site 1 (pLucBΔ) was constructed. As shown in luciferase assay, cotransfection of pLucB, but not pLucA, with p53 expression vector resulted in p53-dose dependent induction of luciferase activities. In contrast, there was no significant increase of luciferase activity after cotransfection with empty vector (EV) or vector expressing p53-R175H (Figure 5F). Furthermore, only the cells transfected with pLucB, but not pLucBΔ showed significant increases of luciferase activity in a p53 dependent manner (Figure 5G). In summary, these data suggest that p53 exerts direct transcriptional regulation of *ELOVL3* through DNA binding between p53 and p53 binding site 1 in ELOVL3 promoter. In addition, the alignment between human and mouse *ELOVL3* promoter sequences showed a high degree of identity of the p53 responsive element (Supplementary Figure 5A), and ELOVL3 is moderately activated upon expression of mouse p53-3KR protein using a luciferase assay (Supplementary Figure 5B, column 2 vs. column 1). Importantly, the activation of ELOVL3 promoter was greatly increased upon mdmx knockdown by mdmx siRNA (Supplementary Figure 5B, column 4 vs. column 3), further validating p53-mediated regulation of ELOVL3.

Finally, to test the regulation of ELOVL3 by p53 in a physiologically relevant context, H460 cells were treated with doxorubicin to induce DNA damage dependent p53 activation. The expression levels of *ELOVL3* were increased as the time of doxorubicin treatment increased, in a similar way to the increase of p53 target gene *p21* (Figure 5H). To demonstrate this regulation was p53 dependent, the control and p53 knockdown H460 cells were prepared through transfection of the control si-RNA (si-ctr) or *p53*-specific si-RNA (si-p53). These cells were further treated with doxorubicin. The increases of *ELOVL3* levels were reduced in *p53* knockdown cells after doxorubicin treatment, compared to the increases of levels of *ELOVL3* in control knockdown cells. As expected, the induction of p53 target gene *p21* was reduced in *p53* knockdown cells after doxorubicin treatment, compared to that of the control knockdown cells (Figure 5I). Taken together, these results demonstrated that *ELOVL3*, a key brown adipocyte gene, is a direct target of p53 transcriptional regulation mediated by p53 responsive sequence in its promoter.

## DISCUSSION

### Mdmx is involved in p53 degradation but not essential

Because of the critical roles of mdm2 and mdmx in negatively regulating p53, the ability to rescue the embryonic lethality of mdm2 and mdmx knockout mice becomes imperative to gauge the extent of loss of functions for p53, particularly for p53 mutants with partial loss of functions [20, 22]. In this study, we were able to rescue *mdmx* knockout mice with *p53 3KR* mutant, a mutant that retains the DNA binding capacity but loses induction of apoptosis, senescence, and cell cycle arrest, highlighting the importance of these p53 dependent functions during embryonic development.

It is well accepted that mdmx represses p53 transcriptional activity through direct binding. However, the role of Mdmx in p53 degradation remains somewhat controversial. On one hand, several studies showed that the Mdm2/Mdmx heterodimer complex is critical for p53 degradation, suggesting that Mdmx is essential for p53 degradation [48-50]. On the other hand, the levels of p53 remain largely unchanged upon Mdmx knockdown in human cancer cells and in the mutant mice expressing an Mdmx mutant lacking its C-terminal ring domain [25, 51]. Our results showed that the protein levels of p53 are modestly increased in the thymus, spleen and testis of *p53*^*3KR/3KR*^*/mdmx*^*-/-*^ mice, consistent to the role that Mdmx participates in p53 degradation. However, the elevated levels of p53 in *p53*^*3KR/3KR*^*/mdmx*^*-/-*^ mice are modest and also tissue-specific (e.g. no effect observed in livers). More importantly, p53 can be further stabilized in response to DNA damage to similar levels in *p53*^*3KR/3KR*^*/mdmx*^*-/-*^ mice, as to wild-type or *p53*^*3KR/3KR*^ mice under the same conditions. Thus, it is likely that Mdmx modestly contributes to p53 degradation by forming the Mdmx2/Mdmx heterodimer but p53 can still be effectively degraded by Mdm2 alone in the absence of Mdmx *in vivo*.

### P53 in switching energy sources and remodeling of adipose tissues

As an important player in cancer biology, p53 not only controls apoptosis, senescence, cell cycle arrest, but also plays important roles in regulating cellular metabolism [52]. It has been suggested that p53 promote cell survival under transient metabolic stress, and p53 is known to lower glucose utilization and promotes mitochondrial respiration and fatty acid oxidation [3, 53]. A complete loss of p53 results in decreased exercise capacity due to repressed mitochondrial activities in mice [54]. Nevertheless, the studies of p53 metabolic functions using p53 knockout mice and the studies using p53 gain-of-function mutant mice were hampered by the rapid tumor formation due to loss of tumor suppression functions of p53. By studying the *p53 3KR* mutant in *mdmx* knockout background, we were able to enhance the metabolic functions of p53 in the absence of tumor formation and in the absence of cell death and growth arrest that normally dominate the phenotypes after p53 activation. Moreover, *p53*^*-/-*^*/mdmx*^*-/-*^ mice failed to show similar skinny phenotypes as observed in *p53*^*3KR/3KR*^*/mdmx*^*-/-*^ mice (Supplementary Figure 6), suggesting the phenotypes associated with *p53*^*3KR/3KR*^*/mdmx*^*-/-*^ are p53 dependent. Consistent with the potential p53 dependent metabolic regulation, we observed increased energy expenditure accompanied with enhanced fatty acid utilization in BAT, and brown remodeling of WAT in *p53*^*3KR/3KR*^*/mdmx*^*-/-*^ mice. Notably, brown remodeling is a process that can be induced due to environmental changes, such as cold challenge, or due to aberrant gene expression. To further support with these observations, we found that *UCP1* and *ELOVL3*, which are often associated with BAT activation and brown remodeling, were upregulated in *p53*^*3KR/3KR*^*/mdmx*^*-/-*^ mice likely due to enhanced p53 3KR activities. This specific effect became more prominent in protection against HFD induced fat accumulation and obesity associated changes. Therefore, p53 is not only critical for metabolic reprogramming in stressed cells, but also for regulating metabolic homeostasis in normal tissues.

Another interesting finding was the decreased macrophage infiltration in *p53*^*3KR/3KR*^*/mdmx*^*-/-*^ mouse adipose tissues, supported by the lacking of crown-like structures (Figure 3F vi and viii) and significant reduction of macrophage marker F4/80 expression (Figure 3G and 3H). Since the recruitment of macrophages into adipose tissue indicates chronic inflammation accompanied with obesity and insulin resistance, decreases in macrophage infiltration further supports the lean phenotype and preserved insulin sensitivity in *p53*^*3KR/3KR*^*/mdmx*^*-/-*^ mice. In addition, apoptotic adipocytes are usually surrounded by macrophages and create crown like structures in adipose tissue. The lack of crown-like structures in eWAT of HFD-fed *p53*^*3KR/3KR*^*/mdmx*^*-/-*^ mice (Figure 3F viii) is consistent to the inability of p53 3KR to induce apoptosis and subsequently lack of apoptosis in adipocytes. Given that increase in lipid oxidation causes increase in reactive oxygen species (ROS) and damage to adipocytes, we propose that p53 has an essential role in regulating adipocyte metabolism: under metabolic stress, such as excess calorie intake (HFD), or increased energy expenditure (during cold exposure), p53 is induced in adipocytes to promote lipid catabolism and potentially also to cause apoptosis; consequently, adipose tissues are replenished with new adipocytes to maintaining adipose tissue homeostasis. In *p53*^*3KR/3KR*^*/mdmx*^*-/-*^ mice, p53 3KR protein is stabilized in the absence metabolic stress, which will promote lipid catabolism in adipocytes, without causing apoptosis in adipose tissue due to the inability of p53 3KR mutant to induce apoptosis. This leads to increased energy expenditure and reduced calorie storage, which is potentially beneficial in preventing obesity. Thus, by using *p53*^*3KR/3KR*^*/mdmx*^*-/-*^ model, we uncovered a unique metabolic control by p53 in adipose tissues, potentially through browning of WAT mediated by a novel p53 target gene, ELOVL3. In summary, our study reveals a potential strategy to combat obesity and the associated increase of cancer risks by targeting Mdmx to modulate p53 functions under physiological conditions [55, 56].

## EXPERIMENTAL PROCEDURES

### Animals

*p53 3KR* mice were described previously [9]. *mdm4* (*mdmx*) knockout mice were provided by Dr. G. Lozano as described [57]. The *mdmx* conditional knockout mice were crossed with *ROSA26-cre* mice to generate *mdmx* knockout heterozygote mice, which were then crossed with *p53 3KR* mice to generate *p53*^*3KR/3KR*^*/mdmx*^*+/-*^ mice. Subsequently, *p53*^*3KR/3KR*^*/mdmx*^*-/-*^ mice were obtained by intercrosses between *p53*^*3KR/3KR*^*/mdmx*^*+/-*^ mice. All mice were maintained on a mixed C57bl6/j and 129Sv/Ev background and housed in a pathogen free facility on a 12-hour light-dark cycle. For the calorimetric analysis, three-month old male mice housed individually for one week in the cages from which food intake, motor activities, O2 input and CO2 output were determined. For the diet-induced obesity experiment, three-month old mice were fed a regular chow diet *ad lib* or a high fat diet (60% kcal fat; Research Diets D12492) for a period of 10 weeks. For the cold challenge experiment, mice were exposed to 4 °C for four constitutive days. All experiments were approved by the Institutional Animal Care and Use Committee (IACUC) of Columbia University.

### Immunohistochemistry

For histological analysis, tissues were fixed in 4% formaldehyde in PBS, embedded in paraffin. The sections (5-µm) were collected and stained with antibody against p53 (CM5, Novocastra, UK) and Cleaved Caspase-3(Asp175) (#9661, Cell Signaling, Danvers, MA, USA), followed by counter staining with hematoxylin.

### Western blot analysis

Whole cell extracts were prepared from tissues by homogenizing in RIPA buffer as described previously [58]. Lysates were separated by SDS-PAGE before being transferred onto nitrocellulose membrane. The membrane was probed with indicated antibody and the recognized proteins were visualized by ECL.

### Quantitation of metabolic phenotypes using biometric cage

Mice were housed individually in metabolic cages to measure the calorimetry, activity, and drinking/feeding intake for a period of one week. Representative data of a period of one day are shown.

### Glucose tolerance test (GTT) assay

The cohorts of mice were fasted overnight. After peritoneal injection of 2g glucose/kg body weight, the blood sugar levels were determined by glucometer (OneTouch UltraMini glucometer from LifeScan, Johnson & Johnson) using blood drop from tail vein, at time points of 15, 30, 60, 90, and 120 minutes.

### Gene expression

To determine the expression levels of gene of interest, total RNA was extracted from cells using TRIzol according to manufacturer’s protocol, which was then used to prepare first strand cDNA by reverse transcriptase. Relative expression levels were determined by quantitive PCR (qPCR) using first strand cDNA and gene specific primers (Supplementary Table 1) according protocol suggested by manufacturer (applied biosystems).

### Cell culture, plasmid generation, transfection and reagent treatment

H1299 and H460 cell lines were cultured in DMEM medium with supplementing 10% (vol/vol) FBS. All the cell lines were obtained from ATCC and are free of mycoplasma contamination. Expressing constructs were made using pTRIPZ vector and stable expression cell lines were established in H1299, subsequently the expression of the integrated genes can be induced by doxycycline. Activation of p53 target genes such as p21, mdm2 was determined by western blot.

### Chromatin immunoprecipitation (ChIP) assay

H1299 cells were transfected with empty vector or expressing construct p53-WT for 24 hrs. The relative occupancy of p53 on potential p53-binding sites of ELOVL3 promoter was evaluated by qChIP assay. Cells were fixed by 1% formaldehyde for 10 minutes at RT and lysed with ChIP Lysis Buffer (50 mM Tris-HCl pH 8.0, 5 mM EDTA, 1% SDS, 1× protease inhibitor) for 10 minutes at 4 °C. After sonication, the lysates were centrifuged, and the supernatants were collected and pre-cleaned in Dilution Buffer (20 mM Tris-HCl pH 8.0, 2 mM EDTA, 150 mM NaCl, 1% Triton X-100, 1× protease inhibitor) by salmon sperm DNA saturated protein A agarose (Millipore, 16-157) for 1 hour at 4 °C. The pre-cleaned lysates were aliquot equally and incubated with indicated anti p53 antibody or control IgG overnight at 4 °C. Saturated Protein A agarose was added into each sample and incubated for 2 h at 4 °C. The agarose was washed with TSE I (20 mM Tris-HCl pH 8.0, 2 mM EDTA, 150 mM NaCl, 0.1% SDS, 1% Triton X-100), TSE II (20 mM Tris-HCl pH 8.0, 2 mM EDTA, 500 mM NaCl, 0.1% SDS, 1% Triton X-100), Buffer III (10 mM Tris-HCl pH 8.0, 1 mM EDTA, 0.25 M LiCl, 1% DOC, 1% NP40), and Buffer TE (10 mM Tris-HCl pH 8.0, 1 mM EDTA), sequentially. The binding components were eluted (1% SDS, 0.1 M NaHCO_3_) and performed reverse cross-link at 65 °C for at least 6 hours. DNA was extracted by PCR purification Kit (Qiagen, 28106). Real-time PCR was performed to detect relative enrichment of p53 on *ELOVL3* gene.

### Electrophoretic mobility shift assay (EMSA)

Highly purified p53 was incubated with a ^32^p-labelled probe (190 base pairs) containing p53-binding element of *ELOVL3* promoter or mutant ELOVL3 promoter in 1× binding buffer (10 mM Hepes, pH 7.6, 40 mM NaCl, 50 µM EDTA, 6.25% Glycerol, 1 mM MgCl_2_, 1 mM Spermidine, 1 mM DTT, 50 ng/µl BSA, 5 ng/µl sheared single strand salmon DNA) for 20 minutes at room temperature (RT). The complex was analyzed by 4% TBE-PAGE and visualized by autoradiography. The probe was obtained by PCR, labelled by T4 kinase (NEB, M0201S) and purified by Bio-Spin column (Bio-Rad, 732-6223).

### Luciferase assay

*ELOVL3* promoter regions (pLucA: control vector containing *ELOVL3* promoter with large deletion; pLucB: containing intact *ELOVL3* promoter and putative p53-binding site; pLucB Δ: containing *ELOVL3* promoter with deletion of the putative p53-binding site) were cloned into pGL3-luciferase construct. The *ELOVL3* luciferase construct was co-transfected with a control renilla construct and a p53-WT or -R175H expressing vector into H1299 cells for 24 hrs. Luciferase activity was measured according to dual luciferase protocol.

### p53 dependent activation of ELOVL3

H460 cells were treated with 1 µM doxorubicin for 12, 24 or 36 hrs. The expression levels of *ELOVL3* and p53 target gene *p21* were determined by qPCR. To test p53 dependent transcriptional regulation, cells were transfected with p53-specific siRNA oligos for 48 hrs to knockdown p53, before the cells were treated with or without 1 µM doxorubicin for 24 hrs to induce p53 expression.

### Statistical analysis

Results were shown as the means ± SEM. Difference was determined by using a two-tailed, unpaired Student *t* test.

## SUPPLEMENTARY MATERIALS FIGURES AND TABLE


