# Supplementary material for: Inhibition of Mdmx (Mdm4) in vivo induces anti-obesity effects
Source: Oncotarget. 2018 Jan 2;9(7):7282–97. doi: 10.18632/oncotarget.23837 (PMC5800902; doi:10.18632/oncotarget.23837)
Supplement: Supplementary file 1 [file oncotarget-09-7282-s001.pdf]

Inhibition of Mdmx (Mdm4) *in vivo* induces anti-obesity effects

SUPPLEMENTARY MATERIALS

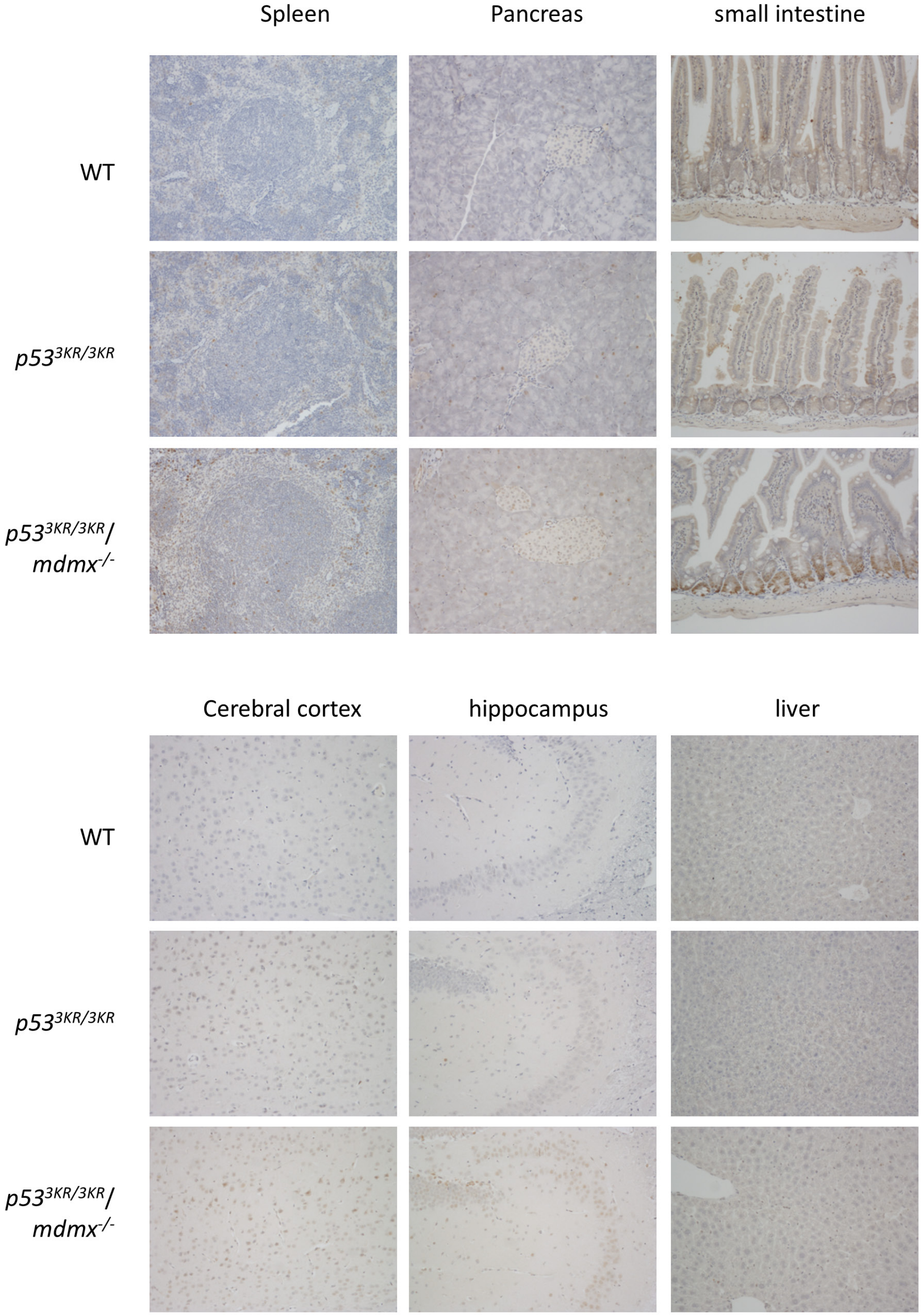

Supplementary Figure 1: Comparison of immunohistostaining of p53 in tissues from wild-type, *p53*$^{3KR/3KR}$, and *p53*$^{3KR/3KR}$/*mdmx*$^{-/-}$ mice without any treatment.

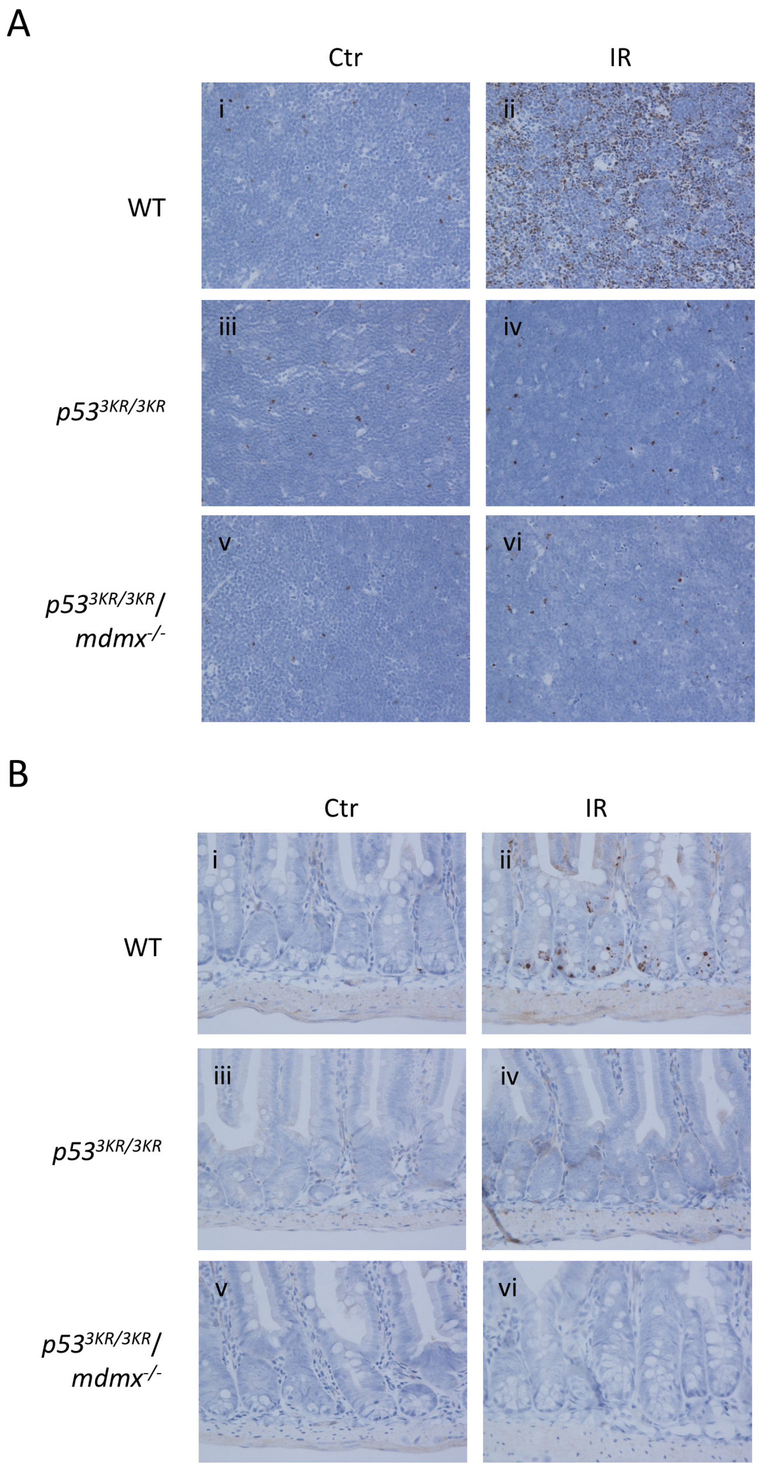

Supplementary Figure 2: Immunostaining of cleaved caspase3 in **(A)** thymus, **(B)** small intestine from (i and ii) wild-type, (iii and iv) $p53^{3KR/3KR}$, (v and vi) $p53^{3KR/3KR}/mdmx^{-/-}$ mice without radiation (i, iii, and v) and treated with ionizing radiation (ii, iv, and vi)**.**

A

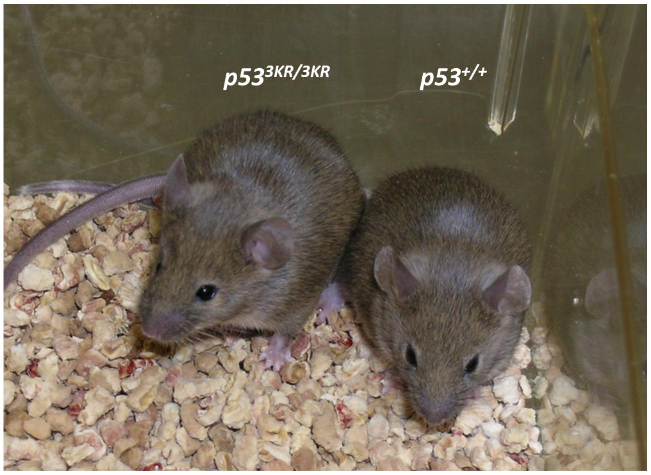

B

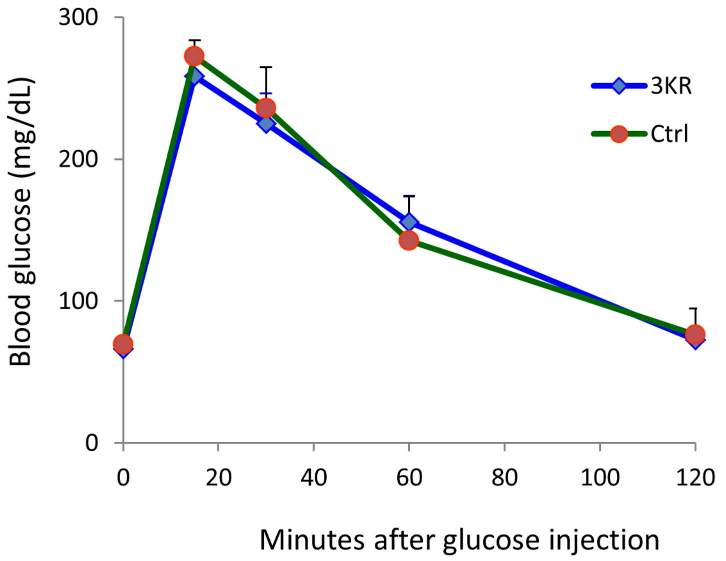

Supplementary Figure 3: (A) Comparison of wild type and p53$^{3KR/3KR}$ mice at one month of age**. (B)** Glucose tolerance test on wild type and ***p53***$^{3KR/3KR}$ mice at 3 month of age.

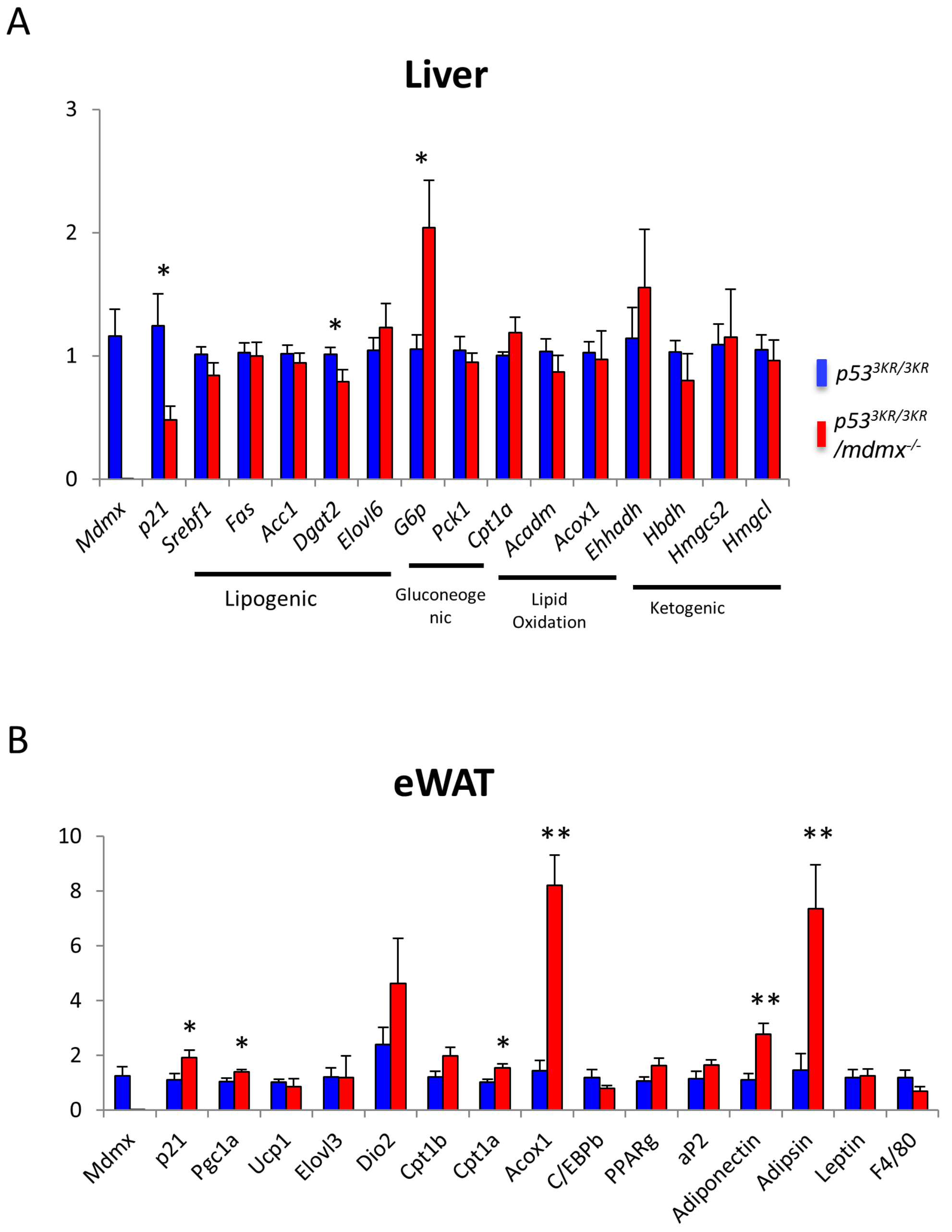

Supplementary Figure 4: Relative expression levels of genes from **(A)** liver (n=10, 6), **(B)** eWAT (n=6, 6) from diet-induced obesity mice same as in Figure 3**.** *p<0.05, **p<0.01. Data are represented as mean ± SEM.

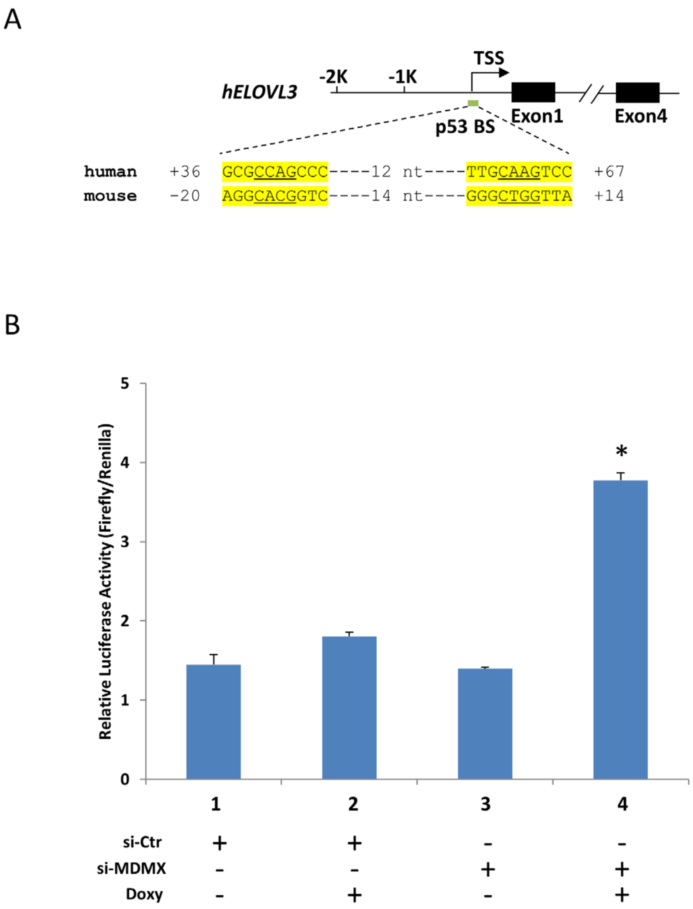

Supplementary Figure 5: (A) Alignment of human and mouse ELOVL3 promoter sequence. Shaded regions represent the p53 binding site. **(B)** Luciferase assay using a mouse p53-3KR inducible H1299 cell line and a human ELOVL3 promoter-driven luciferase expression construct. The cells were first treated with control or *mdmx* siRNA to knockdown mdmx for 4 days, following by induction of mouse p53 in the presence of doxycycline for one day. The *ELOVL3* promoter dependent transcription activities were measured using firefly/renilla overexpression system. *p<0.05, Data are represented as mean ± SEM.

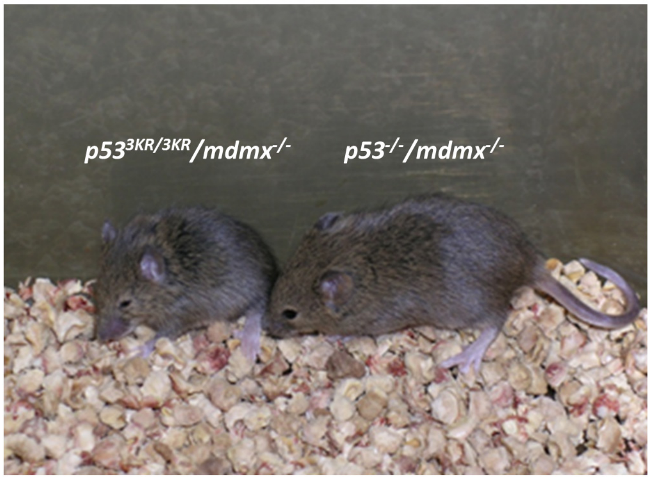

Supplementary Figure 6: Comparison of $p53^{3KR/3KR}/mdmx^{-/-}$ mouse with $p53^{3KR/3KR}$ mouse.

Supplementary Table 1

Genes	Q-PCR Primer sequences
Adiponectin	Forward: GCACTGGCAAGTTCTACTGCAA<br>Reverse: GTAGGTGAAGAGAACGGCCTTGT
Adipsin	Forward: CATGCTCGGCCCTACATGG<br>Reverse: CACAGAGTCGTCATCCGTCAC
aP2	Forward: ACACCGAGATTTCCTTCAAACTG<br>Reverse: CCATCTAGGGTTATGATGCTCTTCA
Atgl	Forward: AACACCAGCATCCAGTTC AA<br>Reverse: GGTTCAGTAGGCCATTCCTC
C/ebpβ	Forward: TGGACAAGCTGAGCGACGAG<br>Reverse: TGTGCTGCGTCTCCAGGTTG
Cpt-1b	Forward: ACCGCAGGAGGAAGGGTAGA<br>Reverse: GCTGAAGAAGGTCTGACGTGC
Dio2	Forward: AGAGTGGAGGCGCATGCT<br>Reverse: GGCATCTAGGAGGAAGCTGTTC
Elovl3	Forward: CCAACAACGATGAGCAACAG<br>Reverse: CGGGTTAAAAATGGACCTGA
F4/80	Forward: CTTTGGCTATGGGCTTCCAGTC<br>Reverse: GCAAGGAGGACAGAGTTTATCGTG
Glut4	Forward: GTGACTGGAACACTGGTCCTA<br>Reverse: CCAGCCACGTTGCATTGTAG
Leptin	Forward: TGACACCAAAACCCTCATCA<br>Reverse: AGCCCAGGAATGAAGTCCA
Mdmx	Forward: TGGAGTCTTCACTGCCAAAA<br>Reverse: GCTGCATGCAAAATCTTCAA
p21	Forward: AGATCCACAGCGATATCCAGAC<br>Reverse: ACCGAAGAGACAACGGCACACT
Pgc1α	Forward: CCCTGCCATTGTTAAGACC<br>Reverse: TGCTGCTGTTCCTGTTTTC
Pparγ	Forward: GCTGTGGGGATGTCTCACAATG<br>Reverse: GTCTTTCCTGTCAAGATCGCCC
Prdm16	Forward: TGGCCTTCATCACCTCTCTGAA<br>Reverse: TTTCTGATCCACGGCTCCTGTGA
Ucp1	Forward: ACTGCCACACCTCCAGTCATT<br>Reverse: CTTTGCCTCACTCAGGATTGG
